# Conscientious Objection in Health Care: Pinning down the Reasonability View

**DOI:** 10.1093/jmp/jhaa029

**Published:** 2020-12-29

**Authors:** Doug McConnell

**Affiliations:** University of Oxford, Oxford, United Kingdom

**Keywords:** conscience, conscientious objection, health care, professional standards, public reason, reasonability

## Abstract

Robert Card’s “Reasonability View” is a significant contribution to the debate over the place of conscientious objection in health care. In his view, conscientious objections can only be accommodated if the grounds for the objection meet a reasonability standard. I identify inconsistencies in Card’s description of the reasonability standard and argue that each version he specifies is unsatisfactory. The criteria for reasonability that Card sets out most frequently have no clear underpinning principle and are too permissive of immoral objections. Card has also claimed that petitioners must justify their positions with Rawlsian public reason. I argue that, although the resulting reasonability standard is principled, it is overly restrictive. I also show that a reasonability standard built on Rawls’ more lenient conception of reasonableness would be overly permissive of objections at odds with professional healthcare standards. Finally, I argue for my favored solution, which bases the reasonability standard on minimal professional standards.

## I. INTRODUCTION

Robert Card’s recently developed “Reasonability View” (2014, 2017a, 2017b) is a significant contribution to the debate over the place of conscientious objection in health care. In his view, conscientious objections can only be accommodated if the grounds for the objection are sufficiently reasonable. In practice, prospective conscientious objectors petition review boards that grant conscientious objector status if they judge that the grounds of the objection meet a reasonability standard (Card, 2014, 321; 2017a, 224).

The Reasonability View would be a major change from the status quo. Authorities that currently accommodate conscientious objections, such as in the United Kingdom and the United States, do not require objectors to justify their objections (Meyers and Woods, 2007; Cowley, 2016). Various steps are taken to minimize the harm that objections cause, such as requiring objectors to refer patients to treating healthcare professionals (HCPs), but the reasoning and beliefs grounding the objection are never assessed. Authorities in other countries, such as Sweden, have no provision to accommodate conscientious objections, whatever their grounds (Munthe, 2016). Both of these approaches to conscientious objection face problems that the Reasonability View can solve. If we accommodate objections whatever their grounds, we will accommodate objections with discriminatory, self-interested, and incoherent grounds that undermine the quality of public health care (Meyers and Woods, 2007; Harries, Stinson, and Orner, 2009). If we rule out objections without assessing their grounds, we will coerce objecting HCPs into going against their consciences, even when they have good reasons to object. The Reasonability View represents a moderate position that promises to uphold the standards of public health care by preventing unreasonable objections *and* protect the consciences of HCPs by accommodating objections based on sufficiently good reasons.

If the Reasonability View is to deliver on these promises, however, its reasonability standard must be principled and neither unfairly permissive nor restrictive of objections. In this article, I identify inconsistencies in Card’s description of the reasonability standard and argue that each version he specifies is unsatisfactory. The criteria for reasonability that Card sets out most frequently have no clear underpinning principle and are too permissive of immoral objections. Card has also claimed that petitioners must justify their positions with Rawlsian public reason. This results in a different reasonability standard that is principled but far too restrictive. Not only would it sometimes demand a higher level of justification from the petitioner than authorities could provide for the healthcare service objected to, the Reasonability View itself would be unable to reach its own justificatory standard. In a first attempt to rescue Card’s view, I develop a more attainable reasonability standard built on Rawls’ principle of reasonableness. However, this reasonability standard places such a high value on pluralism that it is overly permissive of objections grounded in beliefs that are at odds with healthcare values. My second attempt to rescue Card’s view responds to this problem by basing the reasonability standard on Alida Liberman’s (2017) concept of minimally decent health care. On this version of the Reasonability View, conscientious objections grounded in beliefs that are inconsistent with being a minimally decent HCP are unreasonable and should not be accommodated.

## II. THE REASONABILITY VIEW

Card’s view is based on the assumption that the physician’s fiduciary duty to the patient entails a prima facie obligation to provide any legal treatment that the patient requests where that treatment is plausibly in the patient’s interests, consistent with distributive justice, and within the physician’s professional purview.^1^ Therefore, if the physician wants an exemption from his duty, the burden is on him to provide sufficiently good reasons for that exemption. Card’s characterization of the physician’s fiduciary duty is contested. Some claim the fiduciary duty is weaker so that the physician can override it on the basis of his personal moral judgment (Sulmasy, 2017, 25). Therefore, the physician has sufficient authority to make conscientious objections without providing his reasons or having them evaluated. Others believe that the physician’s fiduciary duty is stronger, so that there are no reasons that could justify a conscientious refusal of service (Savulescu, 2006; Savulescu and Schuklenk, 2016). For those who disagree with Card over the strength of the physician’s fiduciary duty, the reasons that the conscientious objector can provide in favor of his objection are irrelevant to whether it should be accommodated and, therefore, the Reasonability View is redundant. For the purposes of this article, however, I assume that Card is correct about the strength of the physician’s fiduciary duty.

If the conscientious objector has to meet a standard of reasonability to have the objection accommodated, clearly, we need to know what that standard is. Card’s reasonability standard builds on a position developed by Meyers and Woods (2007), who claim that we should only accommodate objections that objectors can show are based on sincerely held moral beliefs.^2^ If we do not impose this restriction, HCPs will claim conscientious exemptions from services for relatively trivial reasons (Meyers and Woods, 2007, 20). Objections of trivial importance to the objector should not be sufficient to override the HCP’s fiduciary duty to the patient. Card agrees and incorporates this sincerity requirement in his reasonability standard (Card, 2014, 322–23). However, he argues that sincerity is insufficient for reasonability, because conscientious objections based on sincerely held beliefs can still be unreasonable. Card illustrates this with two kinds of cases. The first kind is where the petitioner’s objection is based on empirical falsehoods. Imagine a physician conscientiously objects to giving children a vaccine on the grounds that the vaccine causes autism and he cannot expose children to that harm.^3^ The physician insists on his belief despite the only study suggesting the link being discredited and a large dataset suggesting that there is no link. The second kind of case is of conscientious objections based on false moral beliefs. Imagine a physician who refuses to treat patients of other races because he sincerely believes that they do not deserve public health care (Card, 2017b, 84). Card’s intuition (which I share) is that the substantive content of the beliefs grounding objections is relevant to whether they should be accommodated; therefore, we need a way of vetting that content (2017b, 93).^4^ Card’s solution is to require those petitioning for a conscientious exemption to convince a review board that their conscientious objections are reasonable. The board “would not simply try to determine whether the medical provider’s belief is genuine . . . but would instead use the reasonability standard” (Card, 2017a, 224). The reasonability standard that Card describes consists of several criteria.

## II. THE REASONABILITY CRITERIA AND THEIR PROBLEMS

Card specifies the criteria for reasonability in most detail in his 2014 article and reiterates them in later articles (2014, 322–24; 2017a, 222; 2017b, 91–92). The reasonability of an objection depends on the beliefs that support it (intrinsic factors) and the particular circumstances in which the objection would be exercised (extrinsic factors). The first intrinsic factor is that conscientious objections must be grounded in genuinely held moral beliefs. This is intended to rule out insincere objections and objections that are of trivial importance to the objector.^5^ The second intrinsic factor is that the beliefs grounding the objection must be consonant with relevant medico-scientific data. This condition rules out objections based on empirical errors, such as believing that vaccines cause autism. These two intrinsic factors are necessary conditions. Extrinsically, the objection cannot cause needless or unjustified harm, fail to provide care in a time-sensitive situation, be discriminatory, or appear purely self-interested.^6^ “The extrinsic factors are not intended to be a list of necessary and sufficient conditions, but should instead be viewed as possessing prima facie weight: there are cases in which the gravity of one or several of these factors may support granting an exemption in one circumstance and not in another” (Card, 2017b, 91). So, roughly, the petitioner needs to show that the beliefs grounding their objection are sincere and align with medico-scientific data. That done, the petitioner also needs to show that, in the circumstances where the objection will be exercised, the benefits of protecting his conscience will not be outweighed by a range of harms that could be suffered by the patient and others.

The first problem with these reasonability criteria is that they are too permissive of objections grounded with immoral beliefs. The problem arises because Card does not make the moral reasonability of the objector’s beliefs a *necessary* condition for granting conscientious objections. Considerations relevant to morality (discrimination, unjustified harm, and self-interest) are incorporated with the extrinsic factors and only given prima facie weight. To illustrate, consider a physician who conscientiously objects to providing a patient analgesia because she sincerely believes that it is immoral to treat patients of other races. Her beliefs are consonant with medico-scientific data because that data only tells us which treatments work and when; it does not resolve normative issues of who should be provided treatments. Therefore, her objection meets the intrinsic criteria and we can move to weigh the prima facie extrinsic factors. The objection is discriminatory and will cause harm to the patient, but this might be outweighed by other circumstantial factors. If we weigh the harm to the patient against that harm to the physician, we find that the physician can withhold pain relief up until the point that it would cause the patient more pain not to receive it than it causes her to provide it. This is clearly unacceptable; the patient should not be exposed to *any* additional harm when an objection is morally unjustified. One might hope that the contribution of other circumstantial factors will block the immoral objection. For example, accommodating a racist refusal of service might cause harm to many in society, and that total harm might clearly outweigh the harm to the physician of going against her conscience. However, we can easily imagine scenarios where the harm to the physician is not outweighed, for example, in societies where the large majority is racist, indifferent, or ignorant. If we treat discrimination, unjustified harm, or self-interest as prima facie extrinsic factors, that entails we accept them in circumstances where their negative effects happen to be outweighed. But, conscientious objections supported by discriminatory or purely self-interested reasons or causing unjustified harm should not be accommodated in *any* circumstances.^7^

Similarly, Card’s criteria are too permissive of conscientious objections made on other grounds that we should want to limit more strictly. Liberman (2017) considers problem cases where objections are based on views of moral wrongness or moral responsibility that are arbitrary or informed by the wrong kinds of consideration. For example, a pharmacist might believe it is morally wrong to use contraceptives on weekdays and/or that she is morally responsible for contraceptives sold on weekdays. We might want to limit objections that are made on grounds that have no clear moral basis (2017, 7). In another kind of case, Giubilini (2016) imagines a physician who attributes to bacteria an unusually high moral status and so objects to administering antibiotics. The possibility of cases like this encourages us to place limits on the substantive conceptions of the good that ground objections. As Card’s reasonability criteria stand, we would have to accommodate all these kinds of objections, as long as the benefits to the objector were not outweighed by the prima facie extrinsic considerations.

All that said, Card should retain a criterion that assesses extrinsic factors in order to rule out objections that would cause disproportionate harm, even if the grounds for the objection are sufficiently sincere, empirically accurate, and moral (assuming a satisfactory assessment of morality can be found). Indeed, it is typical for views that accommodate certain objections to adopt a criterion to prevent disproportionate harm (Meyers and Woods, 2007; Wicclair, 2011, 99–130; Kantymir and McLeod, 2014; Liberman, 2017). However, such a criterion does not thoroughly rule out objections made on immoral grounds, and so it does not do all the work Card needs it to.

## III. PROBLEMS WITH THE THRESHOLDS FOR REASONABILITY

Card also faces difficulties in setting the thresholds for each of his intrinsic reasonability criteria. In order to meet the sincerity requirement, Card suggests that the moral belief grounding one’s objection must be “so central that one can provide evidence that this belief influences important practices in one’s life” (2014, 322). However, it is not clear what would count as sufficient evidence. Card might be happy to let the review board use its judgment; however, that *laissez faire* attitude leaves ample room for bias and inconsistency between boards. For present purposes, I withhold judgment on Card’s threshold for sincerity. This is, in part, because the difficulty in setting a sincerity threshold and assessing sincerity are not problems specific to the Reasonability View. Several accounts depend on being able to assess sincerity and so face this issue (Meyers and Woods, 2007; Kantymir and McLeod, 2014; Liberman, 2017). Furthermore, Card’s view does not depend on any specific version of the sincerity requirement; all he requires is that there be *some* satisfactory version(s) of the sincerity requirement.^8^ If a better version existed, then Card could readily switch to it without undermining the distinctive feature of his view, that is, its assessment of the content of the petitioner’s beliefs.

Card’s assessment of the empirical basis of the petitioner’s objection, on the other hand, is a defining feature of his view. Card’s clearest description of the threshold for empirical reasonability is in response to Jason Marsh. Marsh (2014) pointed out that, if the objector has to convince the review board that the beliefs grounding his objection are *true*, then almost no conscientious objections will ever be granted. Card replies that the objector does not have to convince the review board that the grounds for their objection are true; rather, they just need to provide sufficient evidence of truth.

The evidence for the claim that p is the case is ideally related to the notion that p is true since the stronger the relevant evidence, the more reason there is to believe that p. But evidence and truth are different things. Just as courts assess the evidence in support of a claim according to a certain standard—say, that the preponderance of the evidence supports one’s case—so should [the review] board. (Card, 2014, 321)

So, the relevant standard is truth, but the petitioner only needs to provide enough evidence to the review board to convince them that his beliefs have a reasonable claim to being true. Therefore, there can be multiple conflicting positions, each supported by enough evidence to show they fall within the range of reasonability. Now what are the limits of that range? A couple of Card’s remarks suggest that he sets it quite narrowly. He says that one has to show that the “preponderance of the evidence supports one’s case” (2014, 321) and at another point says that one needs to show that one’s beliefs do not conflict “with the bulk of the relevant clinical evidence” (2017a, 222). Card does not explain why he sets the threshold for empirical reasonability at this level; without such an explanation, it appears arbitrary. If we are to rule out the conscientious objections of HCPs who support relatively marginal empirical views, we need principled grounds for doing so. In the final section, I argue that an appeal to minimal professional standards can potentially justify the threshold that Card suggests.

Christopher Cowley also correctly points out that “this language of truth, and greater likelihood of truth, is most at home in the empirical sciences; it cannot be so directly applied to moral enquiry” (2016, 71). In conflicts between medico-scientific theories, we can usually hope that future evidence will settle the matter. Conversely, conflicts between different conceptions of the good “tend to founder quickly on dogmatic foundational stances with only intuitive authority” (2016, 71), where further data are unlikely to settle the issue. As Card’s criteria for reasonability stand, they avoid these concerns because they only require the objector to demonstrate the reasonableness of the *empirical* beliefs grounding her objection, not her moral beliefs. As I argued above, however, this lack of moral assessment is a problem for Card. If he is to rule out immoral objections, then he has to ask petitioners to show that the beliefs grounding their objections meet a threshold for morality. Therefore, Card cannot dodge Cowley’s concern. Perhaps Card recognizes this bind because, rather than sidestep Cowley’s concern by pointing out that he is only interested in the empirical beliefs grounding an objection, he adjusts his reasonability criteria to address nonempirical beliefs.

## IV. INTELLIGIBILITY AND ARBITRARINESS

In his reply to Cowley, Card (2016) claims that intelligibility and arbitrariness are measures we should use to assess the grounds of objections outside of areas that are settled by empirical evidence. He thereby expands the criteria for reasonability by introducing a necessary assessment of the petitioner’s moral beliefs.^9^ Unfortunately, given the space constraints on the reply, there is not much discussion of how arbitrariness and intelligibility operate in his view. Neither have these considerations been expanded on in subsequent articles as yet. However, as they stand, each condition is inadequate for blocking the range of immoral objections we should want to block. Taking intelligibility first, Card says,

A crucial part of reasonability is that one’s claim is *understandable* by others, and the proposition that a zygote possesses full moral standing at conception is a statement that is *intelligible*, whether one believes it to be true or not . . . There is no empirical evidence that verifies or falsifies the prolife proposition. (2016, 74, my italics)

The first issue here is that “intelligibility” can mean different things. Taken very broadly, to be intelligible is to not speak in nonsense or gibberish. Obviously, this is essential to have an objection accommodated because the review board cannot assess nonsense or gibberish. Once we get over that low threshold for intelligibility, however, we find that nearly everything is intelligible, including fictional stories and immoral actions. Racist beliefs, for example, can be understood (without being condoned) as the expression of innate xenophobia or the result of being a soldier who fought against people of other races. This conception of intelligibility is, therefore, too broad to rule out immoral objections. Card might intend a narrower conception of intelligibility, perhaps one that we use when we signal our agreement with another’s normative assessment. For example, we might find it normatively intelligible that someone attributes fetuses a high moral status, but unintelligible that someone would attribute bacteria a high moral status. However, this runs into another problem—what we find normatively intelligible depends on our cultural background. If one is raised in a social environment where decisions are often justified using religious authority, then one will find such explanations intelligible. If one does not have that cultural background, then such explanations may well be unintelligible. Review boards should not rule out objections just because the petitioner has a different cultural background than the board. Assessment of arbitrariness faces the same problem. Card claims that objections should avoid arbitrariness such as that which infects racist and sexist beliefs (2016, 74). However, there is disagreement over which grounds count as arbitrary. Some believe religious texts ground moral claims, while others see those beliefs as arbitrary. Indeed, some might argue, contra Card, that differences of race and gender are not arbitrary grounds for moral judgments. When a normative position strikes one as intelligible or nonarbitrary that itself does not justify the position, one’s judgment still stands in need of justification. Without justifying the bounds of intelligibility or arbitrariness, Card has not made any progress. Furthermore, it appears Card would treat arbitrariness as a prima facie consideration that might be outweighed, because he says that objections with “a sexist or racist basis, [are] weaker all things being equal” (2016, 73) than objections founded on nondiscriminatory moral or religious beliefs. Therefore, this broadening of the criteria to assess the arbitrariness of the petitioner’s beliefs would still permit conscientious objections based on arbitrary (e.g., racist) beliefs in some circumstances.

In summary, Card’s reasonability standard faces several serious problems. His criteria for reasonability only give prima facie weight to morally relevant factors and so are overly permissive of objections with immoral grounds. The subsequent appeal to standards of intelligibility and arbitrariness does not improve matters. Objections with weak empirical grounds are ruled out more decisively, but the threshold for reasonability—that one’s beliefs must align with the bulk of the evidence—appears arbitrary. As it happens, Card has suggested a theoretical move that would entail a dramatically different reasonability standard. He has claimed that the standard of reasonability comports with the Rawlsian ideal of public reason (2017a, 222–23; reiterated in 2017b, 93). This move solves the problems I have raised above by placing necessary (rather than prima facie) constraints on petitioners’ moral beliefs and by setting a principled threshold for the reasonability of all the beliefs grounding an objection. However, it creates new problems because it sets the threshold for reasonability too high across the board.

## V. A REASONABILITY STANDARD BASED ON RAWLSIAN PUBLIC REASON

To understand the concept of public reason we need to briefly review the theoretical context in which Rawls developed it. Rawls begins with the assumption that citizens in a liberal democracy will develop a plurality of conflicting yet reasonable, comprehensive doctrines (1993, 3–4, 36). Reasonable comprehensive doctrines involve personal religious, cultural, moral, and philosophical beliefs that characterize and organize values so that they are compatible with one another and express an intelligible view of the world (Rawls, 1993, 59). Rawls also assumes that citizens are committed to liberal democracy. For a liberal democracy to function, citizens need to agree on certain constitutional matters and matters of justice, such as regulating the distribution of important resources (Rawls, 1993, 228–29). Citizens’ conflicting personal doctrines create a problem here because different doctrines favor different systems for the constitution and basic justice. Public reason is Rawls’ idea for resolving these conflicts fairly. “Public reason is “public in three ways: . . . it is the reason of the public; its subject is the good of the public and matters of fundamental justice; and its nature and content is . . . open to view” (Rawls, 1993, 213). Public reasons are reasons that all reasonable citizens would endorse; therefore, such reasons can justify rules for the constitution and basic justice, and the enforcement of those rules (Rawls, 1993, 224–26; Rawls, 1997, 771). Card claims that access to public health care is a matter of basic justice and, therefore, that public HCPs “must not solely appeal to their personal, comprehensive doctrine to justify such refusals of care within the institutional structure of medicine but instead must appeal to public reasons” (Card, 2017a, 223). Card outlines the implications of the demand for public reason as follows:

Minimally this means that we can rule out (eg) racist or sexist beliefs as a proper basis for accommodations within the public sphere. Since we can find no good public reason to justify organizing institutions on the basis of rules founded upon the racial or gendered identities of the actors in those institutions . . . we can simply refuse to use these considerations as organizing principles for our societal institutions. *Only objections based on a reasonable conception of the good* can warrant a conscientious exemption within the public institutional structure of medicine. (2017a, 223, my italics)

So, an appeal to public reason provides a way of ruling out objections based on clearly immoral beliefs, such as those that are racist or sexist. It would also rule out objections based on unusual conceptions of the good, such as the belief that bacteria have high moral status or objections based on conceptions of moral wrongness and moral responsibility that reasonable people believed to be arbitrary or improperly informed. In each case, no good public reasons could be found to defend organizing principles based on those kinds of belief. By appealing to public reason, then, Card would avoid the excessive permissiveness of his weaker reasonability criteria. However, as I show in the next section, it sets the threshold for reasonability far too high.

## VI. PUBLIC REASON AND THE THRESHOLD FOR REASONABILITY

The task of finding public reason to support one’s objection is much more challenging than Card acknowledges. It is not enough to provide a justifying reason that is consistent with a reasonable comprehensive doctrine. One must provide a justifying reason that would be agreed on by all reasonable persons. The more diverse the group of reasonable persons, the wider the range of reasonable disagreements, and the more difficult it will be to find public reasons. Rawls is very inclusive about who counts as a reasonable person; reasonable persons believe that all citizens in a constitutional regime should be treated as free and equal, that is, nobody is naturally subject to any other person’s moral or political authority and each is equally situated with respect to this freedom (1993, 55). Reasonable people develop reasonable comprehensive doctrines, that is, doctrines that treat others as free and equal, and recognize sources of permanent reasonable disagreement. The sources of reasonable disagreement are called “the burdens of judgement” and include variable interpretations of complex and conflicting empirical evidence; disagreement over the weight that should be attributed to relevant considerations; a degree of vagueness in our concepts so that they might be applied differently in hard cases; different total life experience shaping different ways of assessing evidence and weighing values; different normative considerations making overall assessment difficult (Rawls, 1993, 56–57). In reasonable disagreements, people continue to recognize each other’s conflicting personal doctrines as reasonable. Therefore, on Rawls’ view, a wide range of doctrines is reasonable, “even though we could not seriously entertain them for ourselves, as we think they give excessive weight to some values and fail to allow for the significance of others” (1993, 59–60). The sources of reasonable disagreement can be contrasted with sources of unreasonable disagreement: prejudice and bias, self- and group interest, blindness and willfulness (Rawls, 1993, 58). People who disagree for these reasons are unreasonable and their views cannot challenge or create public reason.

Card is, therefore, right to say that one needs a reasonable conception of the good to develop a public reason (2017a, 223) because people with unreasonable doctrines will be overruled by reasonable people in the development of public reasons. However, being a reasonable person with a reasonable doctrine only gets you a seat at the table where public reasons are developed. A public reason needs to be supported by all reasonable people; therefore, public reasons are limited by the burdens of judgment (Rawls, 1993, 54). Where reasonable disagreements remain, public reason cannot be found. Therefore, to find public reasons we not only have to avoid appealing to the controversial aspects of our personal doctrines (as Card notes), we also need to avoid appealing to anything that *any* reasonable person could reasonably disagree with.

We are to appeal only to presently accepted general beliefs and forms of reasoning found in common sense, and the methods and conclusions of science when these are not controversial . . . As far as possible, [public reasons] are to rest on plain truths now widely accepted, or available, to citizens generally. Otherwise the political conception would not provide a public basis of justification. (Rawls, 1993, 224–25)

Now that we have a clearer idea of what public reason requires, we can see the unacceptable implications of demanding petitioners provide public reasons to justify their objections. A demand for public reasons does not just rule out the conscientious objections of unreasonable persons, as Card claims. It rules out the conscientious objections of reasonable people with whom others reasonably disagree because, in cases of reasonable disagreement, nobody can support their position with public reason *by definition*. Therefore, public reason is useless to justify any controversial elements of one’s personal comprehensive doctrine; it can only be used to justify compromise positions that lack controversial elements.

To illustrate, consider the case of abortion when the mother’s life is not at risk. The burdens of judgment mean there will be permanent reasonable disagreement over how late in fetal development termination should be permitted. The authorities must decide where to set the stage of fetal development, after which termination is prohibited, somewhere within a reasonable range. They have good reasons for setting it *somewhere* as opposed to *nowhere*, that is, letting individual HCPs decide, because, without guidance, individuals might conduct terminations outside the reasonable developmental range. Furthermore, setting the limit somewhere provides greater certainty about the healthcare services available so that people who use the system can make better informed plans. Wherever the authorities set the latest permissible developmental stage for termination, however, some people will reasonably disagree. Some will disagree with being asked to conduct abortions so late in fetal development, and others will disagree with being asked to abstain from conducting them any later in fetal development. Because these are reasonable disagreements, nobody will be able to find public reasons to support one position over the others. If we insist petitioners provide public reasons in areas of reasonable disagreement, we rule out conscientious objections based on reasonable doctrines.^10^ There would be little point in maintaining a system of review boards when the large majority of conscientious objectors would be unable to satisfy the board (Marsh, 2014). Furthermore, we would inconsistently demand more of petitioners than we do of authorities setting the rules governing the provision of healthcare services. It would be unfair to rule out objections for lack of public reasons and yet oblige HCPs to provide services, such as abortion, which could not be supported with public reasons.^11^ Therefore, Card’s demand that petitioners provide public reasons for their conscientious objections sets the threshold for reasonability too high.

In cases where the petitioner *could* justify her conscientious objection with public reason, that would entail the agreement of all reasonable people so that any pressure to perform the procedure objected to would be an illegitimate use of power. In short, having public reason for one’s objection would justify civil disobedience, not just exemption. This is not surprising when we recall that the point of public reason was to guide the development of rules governing political power. Conscientious objectors are not, typically, attempting to reformulate the set of rules for who can receive what medical procedure (although they may also have that goal); they are merely pursuing an exemption while accepting that the rules will remain the same. So, the standard of public reason is not appropriate to set the threshold for the reasonability of conscientious objections.

There are, however, stronger grounds for requiring *the system* for accommodating or rejecting conscientious objections to be justified with public reason, because such systems set rules that dictate how power can be exercised over citizens in public domains. A version of the Reasonability View that demands public reasons from petitioners is inconsistent because it would not, itself, find support in public reason. The reasonability standard it uses summarily rejects petitions in areas of reasonable disagreement, coercing reasonable HCPs to provide services that go against their reasonable comprehensive doctrines. Reasonable citizens could not agree to such a system, given that it would fail to treat all reasonable citizens as free and equal. If such a system were implemented, it would wield power illegitimately. Of course, it seems improbable that *any* system for dealing with conscientious objections will find support in public reason. There are multiple loci for reasonable disagreement, one being the assumption with which we began, that the physician’s fiduciary duty to the patient entails a prima facie obligation to provide that treatment (under certain conditions). We may, therefore, need to settle for a system that satisfies as many people as possible, stopping short of satisfying all reasonable people. Whichever system we settle on, we should demand that it be internally consistent, that is, not demand a higher justificatory standard from petitioners than it could attain itself.

## VII. THE RAWLSIAN REASONABILITY VIEW

An obvious path to avoiding the difficulties created by the high threshold of public reason is to use Rawls’ much lower threshold for reasonableness.^12^ Rather than justify one’s objection with reasons that all reasonable people would agree with (as required for public reason), one would only need to show that the beliefs grounding one’s objection were part of a reasonable comprehensive doctrine and, therefore, that one’s objection was an expression of reasonable disagreement. This approach would be much more permissive, classifying most of the typical grounds for conscientious objection as reasonable. For example, it would be reasonable to object to the termination of fetuses at any developmental stage (when the mother’s life was not in danger). The Rawlsian approach would rule out conscientious objections with discriminatory grounds because they treat some citizens as less than free and equal and arise from sources of unreasonable disagreement, that is, prejudice, bias, and self-interest and group interest. Therefore, objections based on sexist, racist, or homophobic beliefs, for example, would be deemed unreasonable. Reasonable persons are also sufficiently rational to advance a set of ends (and associated means) consistent with their conception of the good (Rawls, 1993, 50–54). This Rawlsian view can, therefore, rule out internally incoherent objections because reasonable people would not stand by objections that were ends–ends inconsistent or means–ends incoherent. To illustrate, consider Liberman’s case where the physician objects to saving the life of an immoral lobbyist because the physician believes that he would then be responsible for his patient’s future lobbying (2017, 7–8). To hold the physician responsible for the actions of people whose lives he saved entails attributing responsibility so liberally that it would render the concept of responsibility trivial. This would be internally incoherent if the objector’s conception of the good relies on a workable concept of responsibility (as most conceptions of the good do).

The problem this Rawlsian approach to reasonability faces is that it is unclear whether it can rule out objections based on false beliefs, such as the belief that vaccinations cause autism, bacteria have a high moral status, or apparently arbitrary moral principles that change according to the day of the week. These beliefs are nondiscriminatory, could be internally consistent with certain conceptions of the good, and might arise from the burdens of judgment, despite being highly implausible to most people. One might object that the burdens of judgment should not entail that it is reasonable to make just *any* interpretation of empirical evidence, provide *any* weighting for certain considerations, or attribute normative force to *any* consideration. Surely, one might say, it is unreasonable to form beliefs without some minimal standard of reflection; or, it is unreasonable to believe things that are widely held to be false; or, to take up an idea that Card suggests but does not develop, it is unreasonable to hold arbitrary moral principles. The challenge for any of these objections is to set a principled threshold for reasonableness. What we see as insufficiently reflective, false, and arbitrary depends on our own normative perspectives. Appeals to the moral authority of a religious text, for example, seem arbitrary, false, and insufficiently reflective to some and not to others. Whichever approach we take to assessing reasonableness will itself have to be chosen in a principled way or we arbitrarily favor some normative perspectives over others. Rawls explicitly mentions that we should only exclude doctrines as unreasonable if we have “strong grounds based on clear aspects of the reasonable itself. Otherwise our account runs the danger of being arbitrary and exclusive” (1993, 59). Unless a principled threshold can be provided, we should not place any further restriction on who counts as reasonable.

Of course, Rawls was not concerned only with HCPs when setting his threshold for reasonability; he was attempting to provide direction for an inclusive liberal democratic society. In this context, to exclude people as unreasonable was to exclude them from the democratic process and to treat them as less than free and equal citizens. There are good reasons to think we should be more restrictive of those we treat as reasonable HCPs than those we treat as reasonable citizens. In the next section, I draw on Liberman’s recent work (2017) to show how professional ideals can place principled limits on acceptable professional conduct, ruling out the problematic cases of conscientious objection that this Rawlsian approach accommodates.

## VIII. MINIMALLY DECENT HEALTHCARE PROFESSIONALS

“Professionals . . . receive extensive training, and sometimes official licensing, that enables them to provide important public goods and services, and they are in many cases the unique providers of such services” (Liberman, 2017, 3). If they are to fulfil their valuable public roles, HCPs must meet minimum competency standards (Liberman, 2017, 4). Therefore, conscientious objections should not be accommodated if they prevent a professional fulfilling a professional role in a minimally decent way. The public role of HCPs “involves understanding how the body and mind work, diagnosing the ways in which they fail to function well, and helping them function better” (Liberman, 2017, 5).^13^ To be a “minimally decent HCP,” one must display sufficient epistemic, relational, and normative competency to pursue that public role (Liberman, 2017, 4–7). I explain each form of competency in turn.

Professionals will clearly fail to diagnose or treat the patient in ways that will help their mind or body function better if their decisions are based on empirically false beliefs, such as the belief that vaccines cause autism. Therefore, a minimally decent HCP will not make decisions on false empirical grounds, and conscientious objections based on factual errors should not be accommodated (Liberman, 2017, 5).^14^ One might think that the minimally decent HCP should do even better than this and avoid making decisions based on beliefs that are probably false or have relatively little empirical support compared to more mainstream positions. One might argue that the minimally decent HCP should apply mainstream theories where possible because theories with greater empirical support are more likely to be in the patient’s interests. Therefore, we might set the threshold for minimal epistemic competence in medicine higher than Liberman does and specify that the minimally decent HCP should not make decisions based on empirical beliefs that conflict with mainstream empirical positions (where they exist) without a clinical reason for doing so.^15^

Relational competency refers to how the HCP should relate to her patients. According to Liberman, HCPs fail to be minimally competent if they hold discriminatory beliefs (2017, 5–6) or are openly disrespectful to patients (2017, 3). This is because disrespect and discrimination will undermine the effective treatment of patients, undermine public confidence in healthcare professions, and discourage people from seeking medical aid. Therefore, conscientious objections based on discriminatory or disrespectful beliefs will be ruled out for being incompatible with minimally decent health care.^16^

To be normatively competent, minimally decent HCPs must make normative decisions in responsible ways, because patients rely on those decisions. Responsible normative decision-making is informed by reasonable beliefs about moral wrongness and responsibility (Liberman, 2017, 6–7). Liberman claims that conceptions of wrongness and responsibility are unreasonable if they are arbitrary, have no principled basis, or would generate implausible results in analogous cases (2017, 7–8). For example, a doctor would not be a responsible moral decision-maker if he arbitrarily believed it was morally wrong to provide contraception on weekdays but not weekends, or that he was responsible for providing contraception on weekdays but not weekends. Decisions informed by such unreasonable conceptions of wrongness would risk poor health outcomes for patients and so are incompatible with being a minimally decent HCP. Consideration of normative competence would also rule out objections based on conceptions of the good that would compromise patient well-being, such as the physician who objects to providing antibiotics because she thinks that harming bacteria is morally wrong. Obviously, it will be bad for patients if their health care is compromised for the sake of nonsentient creatures, especially in cases where those creatures themselves cause disease. Therefore, conscientious objections based on unreasonable normative positions can also be ruled out. In cases of reasonable normative disagreement, Liberman thinks we should defer to the objector’s own beliefs (2017, 8). So, for example, she thinks it is reasonable for a minimally decent HCP to believe that providing a referral makes her complicit in the future treatment of the patient, but, equally, it is reasonable to think that it does not (2017, 8). Liberman does not specify how she distinguishes a reasonable disagreement from an unreasonable disagreement, but, presumably, disagreements are reasonable when neither of the disputants’ normative positions is at odds with the goals of health care.^17^

## VIIII. JUSTIFYING A CONCEPTION OF THE GOALS OF HEALTH CARE

On Liberman’s view, the goals of health care determine the thresholds for empirical, relational, and normative competence. Clearly, then, this approach depends on being able to justify a conception of the goals of health care, of what counts as objectively good public health care; otherwise, it will arbitrarily rule out conscientious objections grounded in beliefs that conflict with that conception.^18^ A detailed account of objectively good health care goes beyond the scope of this article, but, presumably, good health care involves promoting the patient’s objective health, respect for patient autonomy, and distributive justice (Beauchamp and Childress, 2012). Of course, these parameters leave ample room for debate over what counts as objectively good health, what counts as distributive justice, whether there might be additional healthcare values, and how to prioritize these different values when they conflict. Perhaps, several competing conceptions of the goals of health care will have a sufficient claim to being objectively good health care, in which case the bounds of that set need to be justified.

Another issue that cannot be fully resolved here is whether committing to a conception of objectively good health care entails ruling out religious conceptions of health care and, therefore, religious conscientious objections. One reason for thinking that objectively good health care excludes religious conceptions of health care is that the latter draw on sources that seem arbitrary to those outside the religion, for example, Jehovah’s Witnesses believe blood transfusion to be immoral based on an interpretation of scripture. If religious authorities can define good health care in ways that seem arbitrary to everyone else, then anyone could justify holding any idiosyncratic conception of good health care. If we want to avoid that relativism, we must adopt a conception of good public health care that is justified by appeal to shared healthcare values and rule out competing conceptions of health care that are not grounded in shared values. For example, promoting long, happy, disease-free lives is a mutual value that should shape good health care. Long, happy, disease-free lives are furthered by access to blood transfusions. The value in following particular interpretations of scripture is not shared, and so it does not justify the reduction in life span that would result from eliminating blood transfusions from good public health care. Therefore, we can oblige Jehovah’s Witness physicians to provide blood transfusions in the name of good health care (of course, Jehovah’s Witness patients would still be able to refuse transfusions).^19^ Shared values are necessarily secular values; however, a commitment to shared values does not discriminate against religious conceptions of health care; idiosyncratic secular values that clashed with shared values would also be ruled out, for example, giving bacteria a high moral status. Furthermore, *some* religious grounds for objection may be compatible with publicly justified conceptions of health care. Consider, for example, an objection to abortion based on the belief that the fetus has been given a soul by God and so should be treated as a person. That objection might be publicly justified in secular terms by arguing that health care should further the interests of the fetus because the fetus has a valuable future just like a person (Marquis, 1989). Where religious conceptions of health care align with a justified objective conception of health care, then conscientious objections based on those religious beliefs might be accommodated. For a more detailed discussion of these issues, see Daniel Weinstock (2014) and McConnell and Card (2019).

## X. THE REASONABILITY VIEW AND MINIMAL PROFESSIONAL COMPETENCE

I suggest that Card should adopt Liberman’s approach to set his threshold for reasonability in a principled way. Objections grounded in beliefs that are incompatible with objectively good health care, including discriminatory, disrespectful, empirically false, and normatively unreasonable beliefs, cannot be accommodated because they are incompatible with being a minimally decent HCP. This solution should be attractive to Card because he already appeals to physicians’ professional fiduciary duty to justify his demand that petitioners demonstrate their reasonability. By drawing on Liberman’s argument, he can show that professional ideals do not just require that objecting physicians provide reasons to escape their fiduciary duties; those ideals also place principled restrictions on what counts as a sufficiently good reason. Therefore, Card can solve the problem of defining the reasonability standard in a principled way without any major, additional, theoretical commitments.

At this point, one might wonder whether a version of the Reasonability View based on minimal professional competencies is identical to Liberman’s view and thus redundant. Like Card, Liberman believes that if conscientious objections are to be accommodated, they must be sincere (2017, 3) and must not cause significant harm to patients, even if they are consistent with minimally decent health care (2017, 2). There is, however, one clear distinction between Liberman’s position and a version of the Reasonability View based on minimal professional competence. Liberman specifies that, “Card’s proposal is primarily focused on requiring objectors to defend their positions, while I do not demand that objectors explicitly articulate or publicly defend the reasons for which they act” (2017, 7, note 18). Liberman does not explain why she does not require this of objectors, but her reasons could be normative or practical.

If she does not believe that minimally decent HCPs should face any normative pressure to publicly defend the reasons for which they act, she is being inconsistent. To be a minimally decent HCP, one must uphold one’s fiduciary duty to the patient. If HCPs could exempt themselves from fiduciary duties without providing any reason, the very notion of fiduciary duty would be undermined and the quality of public health care would suffer. Therefore, the minimally decent HCP should have to defend publicly the reasons for his actions when he is asking for an exemption from his fiduciary duty. Perhaps Liberman has practical reasons for not requiring objectors to defend their positions. Clearly setting up and running a system that assesses the grounds for objections involves costs, which might outweigh the benefits of such a system of regulating conscientious objections. This is, in part, an empirical issue that waits on a cost-benefit analysis, but, even if the Reasonability View is more expensive than some alternative systems, it also provides benefits that alternative systems do not. We need to ask ourselves how much we are willing to pay to have a process for dealing with conscientious objections that upholds the quality of the public healthcare system by ruling out unreasonable conscientious objections while also protecting the consciences of reasonable HCPs. Finally, the Reasonability View’s requirement that all petitioners provide the reasons for their objection is a practical strength in that it enforces standards of minimally decent health care particularly thoroughly. Liberman does not explain how her own view would enforce standards of minimally decent health care. However, her discussion suggests that the grounds for objections might only be assessed against the standard for minimally decent health care when disputes arrive in court (Liberman, 2017, 9). If this is true, Liberman’s view would fail to enforce the standards of minimally decent health care in most instances of conscientious objection because patients will rarely take on the burden of legal action. The Reasonability View, in contrast, enforces the standard of minimally decent health care in every instance of conscientious objection and does not place the burden of enforcing that standard on the patient.

I have shown several forms of the reasonability standard to be inadequate. The form that Card has promoted most consistently is too permissive of immoral objections and sets an arbitrary threshold for the extent to which an objection must align with medico-scientific truth. A reasonability standard based on public reason is too restrictive, whereas a reasonability standard based on a Rawlsian conception of reasonableness is overly permissive in the context of professional health care. I have argued that a principled and fair reasonability standard for HCPs should be based on the requirements of objectively good public health care. The resulting Reasonability View accommodates objections when the petitioner can show that the beliefs grounding her objection are sincere and compatible with minimally decent health care, and where the objection would not cause disproportionate harm to others.

## Footnotes

1. In other words, the fiduciary duty does not extend to an obligation to treat patients outside of the places and times where one is employed except in an emergency. For example, dermatologists are not obliged to treat a patient suffering from influenza that they encounter in another hospital ward or on the street, even if they have the training and license to provide the indicated treatment.

2.
Meyers and Woods (2007) do place some further restrictions on the grounds for objections beyond sincerity. However, they focus on structural rather than substantive aspects and they are not clearly guided by a principle. The Reasonability View, in contrast, aims to assess the substantive content of the grounds for objections against the standard of reasonability (discussed below).

3. I borrow this example from Kantymir and McLeod (2014). Card uses an example with a clear empirical error but no obvious moral dimension where a physician refuses to provide pain relief because of a belief that people do not really feel pain (2014, 323). He also uses an example of conscientiously objecting to providing emergency contraception (EC) because of a mistaken belief that EC works after conception (Card, 2011).

4. Card argues that it is insufficient to simply require the objector to refer patients to a nonobjecting physician (2017b, 88–90). A referral condition (alone or combined with a sincerity requirement) reduces the burdens on patients, but it allows physicians to override their fiduciary duty without any excuse. Physicians’ objections, even those grounded on false beliefs, are given precedence over patients’ requests for medically indicated, legal treatments. If the grounds for the objection are clearly wrong, it is not right that *any* additional burden should be put on the patient and other physicians.

5. It will not rule out objections that are morally important to the objector, yet seem trivial to others.

6. Card does not explain why we should assess self-interest by how we expect an objection to appear in practice. A more direct measure of self-interest would assess the objector’s *beliefs*, and, therefore, this should be an intrinsic measure rather than an extrinsic one. Perhaps Card relies on appearances because we cannot trust the objector’s testimony in these cases. Similarly, it would seem that whether an objection was discriminatory or not should depend on the beliefs of the objector rather than on the effects of the objection in the circumstances. In any case, my objection to Card’s criteria below makes concerns at this level of detail redundant.

7. One might think that the necessary requirement that the beliefs be genuine moral beliefs would, at least, block purely self-interested objections because purely self-interested beliefs could not be moral. However, this is not the case because the objector could genuinely believe that their self-interest was moral; perhaps they are a committed ethical egoist. Even if such a position is ultimately contradictory and morally confused, that does not mean that one cannot genuinely take such beliefs to be moral. Therefore, objections grounded on purely self-interested beliefs could meet the sincerity requirement.

8. Some have despaired at finding any workable sincerity requirement and used that as reason in favor of not accommodating any conscientious objections (Schuklenk and Smalling, 2016). I think that this is too quick. There may not be a perfect sincerity assessment, but we could still find one that satisfactorily balances the risk of ruling out sincere objectors and the burden of accommodating insincere objectors.

9. However, he does not appear to recognize this, given that subsequent publications (Card, 2017a, 2017b) continue to specify the original reasonability criteria.

10. A demand that the petitioner provide public reason also conflicts with the threshold for empirical reasonability that Card specifies in his response to Marsh. In that response, he says that one needs to show that the beliefs grounding one’s objection align with the bulk of evidence. However, one’s beliefs could align with the bulk of the evidence and yet reasonable disagreement could remain, because other reasonable people might favor positions with lesser evidential support.

11. Card is wrong when he assumes that there is a public reason to provide abortions because of the improvements in equality for women in societies allowing abortion (2017a, 223). There are reasonable comprehensive doctrines that put a relatively high value on the fetus and so reasonably disagree with abortions on that basis.

12. Occasionally, it seems that this is the standard to which Card really means to appeal and not public reason at all. For example, he says, “as Rawls argues, in the public sphere we can acceptably require that individuals act on the basis of reasonable conceptions of the good” (2016, 73–74).

13. Liberman does not describe her conception of the role for public health care in any detail or attempt to justify it. I return to this issue below.

14. Epistemic competency is slightly more complex than Liberman acknowledges. It would be professionally acceptable to act on a false belief if one had taken the steps a professional should take to try and discover the truth. Time pressure might preclude a conclusive diagnosis before providing treatment, and certain cases might be beyond the current knowledge of the field.

15. This higher standard of epistemic competence for minimally decent HCPs comes very close to Card’s claim that the empirical beliefs grounding petitioners’ objections should not conflict “with the bulk of the relevant clinical evidence” (2017a, 222). Importantly, the appeal to professional competence provides a principled basis for this threshold that was lacking on Card’s account.

16. Here, the conditions for minimally competent professionalism provide reasons to avoid discriminatory beliefs in addition to Rawls’ reasons for avoiding discriminatory beliefs. For Rawls, reasonable people avoid discrimination because we must treat each other as free and equal for the proper functioning of liberal democracy. Typically, we will be committed to both a medical profession and liberal democracy, so both reasons apply.

17. For Liberman, normative reasonableness depends on whether one’s beliefs align with the goals of health care. She thereby avoids the arbitrariness of Card’s position where mere intelligibility was thought to mark normatively reasonable beliefs.

18. If there is no such a thing as objectively good health care, then the effort to limit conscientious objections with a standard of minimally decent health care will be fundamentally misguided.

19. It might seem unfair to oblige those who cannot ground their conceptions of health care in publicly recognizable values to pay for a public healthcare system that, in their view, wastes money on immoral services and demands that physicians provide those services. However, as long as the public healthcare system sufficiently supports their values, it will remain a worthwhile compromise. If it turned out that there was very little overlap in people’s conceptions of good health care, then compromise would no longer be worthwhile and it would be better to abandon a healthcare system that tries to satisfy everyone in favor of a plurality of systems where people pay for the healthcare system that best aligns with their values.
